# Association between Radiologists' Experience and Accuracy in Interpreting Screening Mammograms

**DOI:** 10.1186/1472-6963-8-91

**Published:** 2008-04-25

**Authors:** Eduard Molins, Francesc Macià, Francesc Ferrer, Maria-Teresa Maristany, Xavier Castells

**Affiliations:** 1 Evaluation and Clinical Epidemiology Department, Hospital del Mar, Passeig Marítim, 25-29, 08003 Barcelona, Spain; 2 Radiology Department, CRC-Hospital del Mar, Passeig Marítim, 25-29, 08003 Barcelona, Spain

## Abstract

**Background:**

Radiologists have been observed to differ, sometimes substantially, both in their interpretations of mammograms and in their recommendations for follow-up. The aim of this study was to determine how factors related to radiologists' experience affect the accuracy of mammogram readings.

**Methods:**

We selected a random sample of screening mammograms from a population-based breast cancer screening program. The sample was composed of 30 women with histopathologically-confirmed breast cancer and 170 women without breast cancer after a 2-year follow-up (the proportion of cancers was oversampled). These 200 mammograms were read by 21 radiologists routinely interpreting mammograms, with different amount of experience, and by seven readers who did not routinely interpret mammograms. All readers were blinded to the results of the screening. A positive assessment was considered when a BI-RADS III, 0, IV, V was reported (additional evaluation required). Diagnostic accuracy was calculated through sensitivity and specificity.

**Results:**

Average specificity was higher in radiologists routinely interpreting mammograms with regard to radiologists who did not (66% vs 56%; p < .001). Multivariate analysis based on routine readers alone showed that specificity was higher among radiologists who followed-up cases for which they recommended further workup (feedback) (OR 1.37; 95% CI 1.03 to 1.85), those spending less than 25% of the working day on breast radiology (OR 1.49; 95% CI 1.18 to 1.89), and those aged more than 45 years old (OR 1.33; 95% CI 1.12 to 1.59); the variable of average annual volume of mammograms interpreted by radiologists, classified as more or less than 5,000 mammograms per year, was not statistically significant (OR 1.06; 95% CI 0.90 to 1.25).

**Conclusion:**

Among radiologists who read routinely, volume is not associated with better performance when interpreting screening mammograms, although specificity decreased in radiologists not routinely reading mammograms. Follow-up of cases for which further workup is recommended might reduce variability in mammogram readings and improve the quality of breast cancer screening programs.

## Background

Breast cancer screening shows wide interobserver (radiologist) variability in the interpretation of screening mammograms [1-3]. This variability depends, among other factors, on the protocols for mammogram reading, the specific characteristics of each patient and breast and, to a large extent, on the radiologist's experience. Radiologists have been observed to differ, sometimes substantially, both in their interpretations of mammograms and in their recommendations for follow-up. Therefore, variability in mammogram reading may adversely affect the quality of screening programs by affecting recall rates, which may be low (undetected tumors or diagnostic delay) or high (provoking anxiety in women, false positives, and increased costs) [4-6].

Attempts have been made to explain variability among radiologists by experience-related factors, such as annual reading volume [7-11]. However, few studies have analyzed in depth and integrated into a single analysis several possible predictive factors related to radiologists' experience that could determine probable causes of the variability observed in mammogram interpretation (beyond annual reading volume) and that could help to improve the quality of screening programs.

The aim of this study was to determine the extent to which a series of experience-related factors affects the accuracy of mammogram readings.

## Methods

### Ethical issue

This study will follow the national and international guidelines stated at the Declaration of Helsinki and, furthermore, it will comply with the legal procedures regarding rules of data confidentiality (Law 15/1999 of December the 13th, about Personal Data Protection [LOPD]).

### Mammogram selection

A random sample of 200 mammograms from asymptomatic women aged 50 to 64 years old who had participated in the first and second rounds of a population-based breast cancer screening program in Barcelona City (Catalonia, Spain) was selected. The programme, which began in 1995, was based on the European Guidelines for Quality Assurance in Mammographic Screening [12] and its results met the Europe Against Cancer standards. All mammograms were located at the same radiology unit and readings were performed by the same team of radiologists. All mammograms were read by two radiologists and, when double readings led to different assessments, a third radiologist served as a tie breaker.

A total of 33,435 mammograms were stratified so that the sample included the four possible results of screening: true negatives, true positives, false negatives, and false positives. These results were validated by comparing the original interpretation obtained in the screening program with the result of the mammogram performed in the following round (2 years later). Histological confirmation was available in all women with a final diagnosis of cancer (both carcinoma in situ and invasive carcinoma).

Of the 200 mammograms selected, 30 (15%) corresponded to women with a definitive diagnosis of cancer (14% true positives, 1% false negatives). The remaining 170 mammograms (85%) corresponded to women with a definitive result of absence of cancer (55% true negatives, 30% false positives by recall). For each participant, double-view mammograms were taken (craniocaudal and mediolateral oblique), with a total of four films per participant. All mammograms complied with the following minimum quality criteria: breast situated centrally with the nipple in profile, visualization of all the breast tissue, the pectoral muscle shadow reached the nipple level, the nipple was seen in profile, and the inframamammary angle could be visualized. We excluded a small number of mammograms not meeting these criteria, as well as women requiring more than one film in one of the views, those who had undergone plastic surgery, those with breast implants, and women with radiopaque skin markers on the breast.

Original films (not copies) were always used. All the mammograms were obtained with a standard film-screen technique (Thosiba SSH 140 A and Bennett Trex Medical) using Agfa Mamoray-HT film.

### Radiologists

A random sample of 28 radiologists from the radiology services of distinct health centers in Spain (general hospitals, district hospitals and primary care centers) was selected.

Before beginning data collection, the radiologists were asked if they routinely interpreted mammograms. Depending on their responses, the radiologists were then divided into two groups. The first group included 21 radiologists routinely reading mammograms but with different amounts of experience while the second group included seven radiologists who read mammograms infrequently or who were medical residents in radiology (radiologists not routinely interpreting mammograms).

### Experience-related variables

To determine radiologists' experience in mammograph interpretation, the 21 routine readers were administered a questionnaire designed after a literature review of the possible factors related to radiologists' self-reported experience [11]. Telephone interviews were performed by one of the project's researchers, with prior agreement from participating radiologists. The items referred to routine practice in mammogram interpretation during the year prior to participation in the study. The following experience-related factors were taken into account:

#### Annual reading volume

This variable included both screening and diagnostic mammograms. Annual volume was calculated on the basis of the number of readings made per week, bearing in mind holiday periods and rotations

#### Consultations

Radiologists were asked whether they routinely (frequently) consulted with other radiologists when interpreting mammograms. This variable is an indicator of whether the mammogram reading was performed individually or as a team.

#### Years of experience in reading mammograms

The number of years of experience reading both diagnostic and screening mammograms was evaluated without taking into account years of specialist practice.

#### Radiologists' age

Age at interview (as a proxy variable of experience).

#### Focus on breast radiology

The percentage of working hours included the percentage of time devoted to breast radiology, both mammograms and other diagnostic techniques, during radiologists' working hours.

#### Feedback

Radiologists were considered to obtain feedback when they worked in a team with a protocol for the follow-up of all women in whom they recommended further workup after the screening test (imaging tests or invasive procedures).

### Reading procedure

Given that the aim was to reproduce as far as possible normal mammogram reading practice, the 28 radiologists independently read the set of 200 mammograms at their workplace. For each breast, the radiologists provided information on the following variables: result, breast density (from less dense to more dense), lesional pattern (nodular, distorting fibrous, mixed, calcified, and parenchymatous asymmetry) and location of the lesion. The results of readings were reported according to the Breast Imaging and Reporting Data System (BI-RADS) [13,14]. In the case of more than one lesion in the same breast, only the most severe lesion was reported. At no time were previous mammograms available to radiologists for comparison while interpreting films.

At the beginning of the study, a session was held with all the radiologists to unify the criteria for mammogram data. The radiologists indicated the results in a standard data collection form that included the norms for completion explained in the initial session.

The participating radiologists were blind to both the study design and the proportion of cancers in the sample, although they were informed that cancer cases were oversampled.

### Statistical Analysis

To calculate sensitivity and specificity, mammograms were considered positive (women were recalled for additional investigations) when classified as BI-RADS III, 0, IV or V. Readings were considered negative when they were classified as BI-RADS I or II. A single BI-RADS category was determined for each woman, based on the most malignant of the two breasts.

The area under de the ROC curve (AUC) was evaluated to compare the 21 radiologists routinely interpreting mammograms and the seven radiologists who did not routinely interpret mammograms.

For the univariate analysis, sensitivity and specificity were evaluated in the 21 routine readers according to each experience-related variable, stratified into two levels with a cut-off indicating presumably less and presumably more experience in mammogram reading. The statistical significance of differences in sensitivity, specificity and accuracy between the two levels of routine readers, was determined by generalized score tests trough marginal models (link logit).

Sensitivity, specificity and accuracy were then modeled by use of multivariate logistic regression estimated through the method described in detail by Smith-Bindman et al [15]. Moreover, a global measure of accuracy was calculated; the radiologist was assumed to be accurate when mammograms from women with cancer were classified as positive and those from women without breast cancer as negative. These models were adjusted by all the experience-related variables. Because of their characteristics, the seven radiologists not routinely interpreting mammograms were excluded from this regression. Given that 15% of the women in the sample had cancer and 85% were cancer-free, to estimate accuracy weights were used to assign equal importance to interpretation of mammograms from women with and without cancer. The analysis took into account the correlation due to the radiologists' consistent interpretation across the 200 films. Therefore, marginal models were estimated based on generalized estimating equations (GEE). The analysis was performed through link logit and an exchangeable structure in working correlation matrix. The GENMOD procedure of SAS 9.1 was used.

## Results

Twenty-eight radiologists read the same 200 screening mammograms, representing a total of 5587 readings (13 lost readings). Based on the interpretations of these radiologists, the false-positive rate was 36% (1728 of 4750) and the false-negative rate was 16% (132 of 837). Therefore, the average sensitivity was 84% (705 of 837) (range, 63–97%) and the average specificity was 64% (3022 of 4750) (range, 34–85%) (Table 1).

**Table 1:** Classification of readings performed by the 28 radiologists following the Breast Imaging and Reporting Data System (BI-RADS).

Result	Readings in women with cancer		Readings in women without cancer		Total readings	
	n	%	n	%	n	%
Negative						
BI-RADS I and II	132	15.8	3022	63.6	3154	56.5
Positive						
BI-RADS III	84	10.04	959	20.19	1043	18.67
BI-RADS 0	151	18.04	454	9.56	605	10.83
BI-RADS IV	325	38.83	297	6.25	622	11.13
BI-RADS V	145	17.32	18	0.38	163	2.92
Total	837	100.00	4750	100.00	5587	100.00

3 readings lost in women with cancer

10 readings lost in women without cancer

13 readings lost in total

The seven radiologists not routinely interpreting mammograms showed an average sensitivity of 84% (177 of 210) (range, 63–97%) and an average specificity of 56% (671 of 1189) (range, 34–69%) while the 21 routine readers showed the similar average sensitivity of 85% (531 of 627) (range, 63–97%) (p = .999) but a higher specificity of 66% (2351 of 3561) (range, 51–85%) (p < .001). The global measure of accuracy revealed that 61% (848 of 1399) of readings were correctly classified in the group of radiologists not routinely interpreting mammograms compared with 69% (2882 of 4188) in the groups of routine readers (p < .001) (data not shown).

The 21 routine readers had a mean age of 47 years (range, 40–60 years), had 12 years' experience of reading mammograms (range, 4–22 years), had read an average of 5773 mammograms in the year prior to participating in the study (range, 1890–13230 mammograms), and spent an average 56% of their working hours on breast disease (range, 15–100%). Eighty-one percent (17 of 21) of the radiologists routinely consulted colleagues and 86% (18 of 21) routinely obtained feedback on cases for which they recommended further workup (data not shown). Given the characteristics defining the group of radiologists not routinely interpreting mammograms, experience-related variables were not evaluated in these seven radiologists.

**Figure 1:**
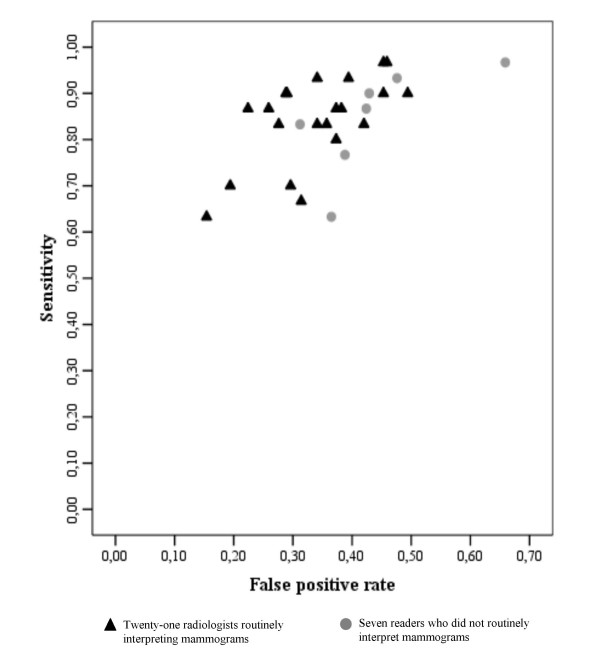
**True-positive rate (sensitivity) of the 28 radiologists versus the false positive rate (1-specificity).** Rates not adjusted for patient variables.

**Figure 2:**
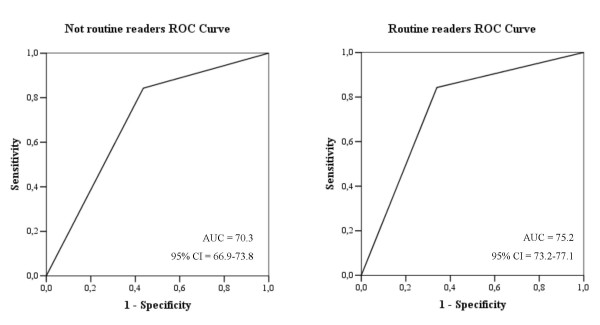
Empirical ROC curves of the seven radiologists not routinely interpreting mammograms and of the 21 routine readers.

Higher sensitivity was often associated with a higher false-positive rate for the 28 radiologists (Figure 1). Given the limited number of cases of cancer and of non-cancer in the sample, these were susceptible to small variations in classification during mammogram reading – hence the wide variability. The AUC for routine readers and radiologists not routinely reading mammograms were evaluated in 70.3 (95% CI 73.2 to 77.1) and 75.2 (95% CI 66.9 to 73.8) respectively, but the greatest difference seems to be observed in the fraction of false-positives in the interval 0%–20% (Figure 2).

When routine readers only were considered, those spending more than 25% of their working day on mammogram reading showed higher sensitivity (86% vs 78%, p = .019) but lower specificity (65% vs 70%, p < .0001). In contrast, those consulting with colleagues showed lower sensitivity (83% vs 90%, p = .036) and higher specificity (67% vs 62%, p < .0001), and those aged more than 45 years old also showed lower sensitivity (81% vs 90%, p = .007) and higher specificity (68% vs 58%, p < .0001) (Table 2).

**Table 2:** Association among experience-related variables in the 21 radiologists routinely interpreting mammograms (univariate analysis)

	**P Sens.**	**Sensitivity**	**P Spec.**	**Specificity**	**Mean AUC**	**Mean AUC90**
Annual volume of mammogram Reading	0.193		0.170			
More than 5,000 mammograms		0.85		0.67	91.66	72.13
Less than 5,000 mammograms		0.83		0.65	83.56	44.45
Feedback	0.239		0.213			
Yes		0.85		0.66	85.71	50.76
No		0.81		0.64	80.66	34.98
Percentage of time spent on breast radiology	0.019		<.0001			
Less than 25%		0.78		0.70	82.36	36.96
More than 25%		0.86		0.65	85.27	49.72
Years of practice reading mammograms	0.116		0.166			
Less than 10 years		0.88		0.65	85.60	47.45
More than 10 years		0.82		0.67	84.49	48.83
Do the radiologist usually consult with other radiologists when reading?	0.036		<.0001			
Yes		0.83		0.67	84.47	48.11
No		0.90		0.62	87.18	50.18
Radiologist' age	0.007		<.0001			
More than 45		0.81		0.68	84.06	48.87
Less than 45		0.90		0.58	87.96	47.34

P Sens., P. Spec. = Chi-square of the score test for Type 3 GEE analysis. Mean AUC = Mean area under the empirical receiver operating characteristic curve. Mean AUC90 = Mean area under the ROC curve con una specificity fixed at 90% of the empirical receiver operating characteristic curve.

**Figure 3:**
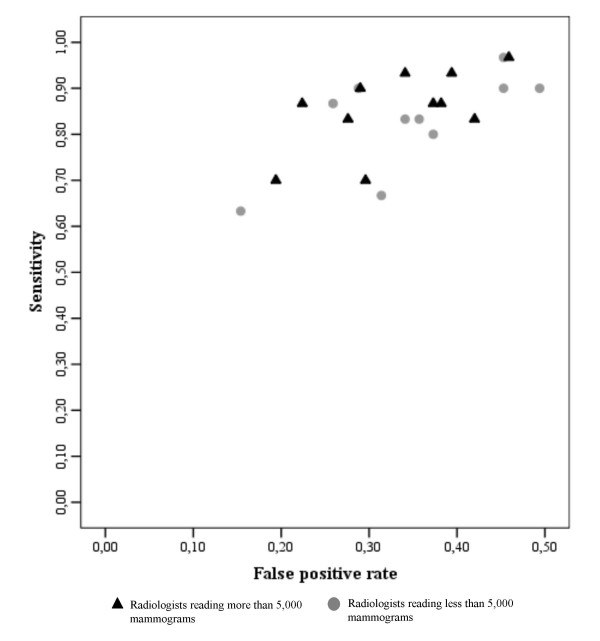
**True-positive rate (sensitivity) of the 21 routine readers versus the false positive rate (1-specificity) distinguishing between radiologists who read less than 5,000 mammograms per year and those who read more.** Rates not adjusted for patient variables.

The variable of annual reading volume showed no significant differences between radiologists reading more than 5,000 mammograms annually and those reading less than 5,000 mamograms annually. No differences were found in sensitivity (p = 0.193) or in specificity (p = 0.170). No patterns were observed when we compared this variable through the representation of sensitivity versus the fraction of false-positives (Figure 3).

The multivariate model was used to evaluate the 21 radiologists routinely interpreting mammograms. The only measure showing statistically significant diffearences was specificity, which was higher in radiologists obtaining feedback (OR 1.37; 95% CI 1.03 to 1.85), in those devoting less than 25% of their working hours to mammogram reading (OR 1.49; 95% CI 1.18 to 1.89), and in those aged more than 45 years (OR 1.33; 95% CI 1.12 to 1.59). The remaining variables (annual reading volume, years of experience in reading mammograms and consultation with colleagues) showed no influence on sensitivity, specificity or accuracy (Table 3).

**Table 3:** Associations among experience-related variables in the 21 radiologists routinely interpreting mammograms and sensitivity, specificity and overall accuracy (multivariate analysis).

	**Sensitivity**			**Specificity**			**Accuracy†**		
			
	**Adj. OR**	**95% CI**	**P**	**Adj. OR**	**95% CI**	**P**	**Adj. OR**	**95% CI**	**P**
Annual volume of mammogram Reading									
More than 5,000 mammograms	0.93	(0.56 to 1.54)	0.780	1.15	(0.98 to 1.35)	0.090	1.06	(0.90 to 1.25)	0.450
Less than 5,000 mammograms	1.00	(reference)		1.00	(reference)		1.00	(reference)	
Feedback									
Yes	0.98	(0.43 to 2.27)	0.957	**1.37**	(1.03 to 1.85)	0.029	1.19	(0.89 to 1.56)	0.243
No	1.00	(reference)		1.00	(reference)		1.00	(reference)	
Percentage of time spent on breast radiology									
Less than 25%	0.70	(0.36 to 1.37)	0.295	**1.49**	(1.18 to 1.89)	0.001	1.10	(0.87 to 1.38)	0.438
More than 25%	1.00	(reference)		1.00	(reference)		1.00	(reference)	
Years of practice reading mammograms									
Less than 10 years	1.40	(0.84 to 2.34)	0.193	1.04	(0.89 to 1.21)	0.640	1.14	(0.97 to 1.34)	0.102
More than 10 years	1.00	(reference)		1.00	(reference)		1.00	(reference)	
Do the radiologist usually consult with other radiologists when reading?									
Yes	0.61	(0.29 to 1.30)	0.202	1.04	(0.84 to 1.28)	0.717	0.89	(0.71 to 1.11)	0.300
No	1.00	(reference)		1.00	(reference)		1.00	(reference)	
Radiologist' age									
More than 45	0.56	(0.31 to 1.01)	0.053	**1.33**	(1.12 to 1.59)	0.001	1.00	(0.83 to 1.19)	0.971
Less than 45	1.00	(reference)		1.00	(reference)		1.00	(reference)	

Adj. OR: Adjusted odds-ratio; 95% CI: 95% confidence interval; P: Chi-square probability

† Correct diagnosis of both cancer and absence of cancer on recalling women for additional investigations.

## Discussion

In the present study, wide variability in radiologists' interpretations of the sample of mammograms was observed. The group of radiologists not routinely interpreting mammograms showed no differences in average sensitivity in mammogram interpretation compared with routine readers but showed significantly less specificity and accuracy. Of the various experience-related factors used to evaluate this variability, annual reader volume was only important when radiologists not routinely interpreting mammograms were compared with routine readers, the latter showing greater specificity and accuracy. In contrast, no significant differences in sensitivity, specificity or accuracy were found among routine readers between those reading less than 5,000 mammograms per year compared with those reading more than 5,000 films. When the remaining experience-related variables were incorporated into a multivariate model, obtaining feedback on cases for which further workup was recommended increased specificity.

To guarantee the quality of population screening programs, substantial efforts have been made during the last decade to understand the role played by radiologists' experience in the variability of screening mammogram interpretations, as well as to identify the radiologist-associated factors determining accuracy. One of the factors considered most important is annual reading volume. In 1998, Elmore et al [11] observed that the annual volume did not significantly influence the recommendation for workup but concluded that radiologists interpreting relatively few mammograms each year, even over many years, may not be sufficiently experienced to obtain high levels of sensitivity and specificity. Kan et al [10] demonstrated that a minimum of 2,500 annual readings guaranteed a better cancer detection rate.

Since 1998, two distinct lines of argument can be discerned: Esserman et al [16] and Smith-Bindman et al [15] concluded that the quality of mammogram readings could be improved by increasing annual reading volume, while Beam et al [17] and Barlow et al [8] reported that reading volume was not an important variable and that radiologists' interpretative performance is a multifactorial process in which a large number of factors play a role. A recent report by the Institute of Medicine containing an exhaustive review of the literature had no been able to demonstrate a clear relationship between volume alone and accuracy [18]. Our opinion is that, unfortunately, in the attempt to guarantee the quality of population screening programs, the study of variability in mammogram readings has been excessively simplified by evaluating the role played by the variable of annual reading volume, with fairly arbitrary cut-off values, beyond which greater accuracy would be achieved.

Our results, like those of other studies, cast doubt on the major role that has been assigned to the variable of annual reading volume as an indicator of radiologists' experience. As in other variables, we observed a positive association between reader volume and accuracy when comparing the group of radiologists not routinely interpreting mammograms with the group of routine readers (established on the basis of the recommended number of 5,000 mammogram readings annually by the European Guidelines [12], the National Health Service in the United Kingdom [19] or Esserman et al [16]). However, we found no significant differences between the two levels of routine readers in either the univariate or the multivariate analyses. Therefore, in addition to questioning the importance of volume, we highlight the role played by other experience-related variables.

According to the results of the multivariate analysis in the present study, one of the most important factors determining experience is feedback (OR 1.37; 95% CI 1.03 to1.85), since it allows radiologists to perform a self-evaluation and become aware of the accuracy of previous readings. Moreover, we believe that the design of screening programs should take this factor into account. The other two significant variables found in this study, focus on breast radiology and radiologists' age should be interpreted conjointly because these variables could show a certain degree of colinearity. Thus, a radiologist aged more than 45 years old spending less than 25% of the working day on breast radiology could correspond to the profile of a highly accurate reader.

Since we found no significant differences in sensitivity between the group of radiologists not routinely interpreting mammograms and the group of routine readers, we believe that sensitivity could present a certain ceiling effect, inherent to the experience-related factors studied to date. This result had previously been discussed in an article explaining how mammography sensitivity has not changed for decades [20,21]. Therefore, we believe that sensitivity is not an appropriate measure to evaluate accuracy, at least not in studies based on mammography samples. We also used the area under the ROC curve, at a specificity of 90%, which allowed us to rank the 28 radiologists according to performance (data not shown). However, for the multivariate analysis, because of correlated data, and given that our objective was to evaluate average accuracy (rather than the individual effect of each radiologist on accuracy), the analysis that we believe optimal was based on marginal models based on generalized estimation equations.

We emphasize our study design because, in a sample of screening mammograms, in which selection of thousands of mammograms and hundreds of radiologists is not feasible and in which cancer cases are necessarily oversampled (bearing in mind that the incidence of breast cancer is approximately 3–8‰ in an incident screening round), the composition of the sample is a key factor for understanding the results obtained. These results depend basically on the proportion of true positives, true negatives, false positives, and false negatives chosen from the program to compose the sample. This composition was chosen according to criteria published by Kerlikowske et al [22]. In this sense, given that the percentage of mammograms with uncertain diagnosis in our study was high, we found a large number of false positives and false negatives and consequently the average sensitivity and specificity were only 84% and 64% respectively, which is substantially lower than the sensitivity and specificity expected in a screening program.

Precisely because we chose a sample not representative of the population, a possible limitation of our study can be attributed to contextual bias. To evaluate the extent to which sensitivity and specificity were influenced by the sample, we performed an *ad hoc *analysis using only the 138 mammograms with a true positive and true negative result, and found that sensitivity did not vary, but that specificity was increased from 64% to 77%. Thus, we justify the study design based on a sample of mammograms by the difficulty of performing a prospective study in which recruitment of a sufficiently large number of radiologists to guarantee adequate statistical power would be difficult.

In addition to experience-related factors, variability is also explained by differences in organization and protocols for reading mammograms, which are not homogeneous in all countries [23,24]. In Europe, screening programs are population-based, publicly financed and adhere to European Guidelines that guarantee the quality of the process [12,25], while in the USA, financing and organization are managed basically by private insurance. However, the characteristics of the protocol should also be taken into account, in which, based on mammography quality, there are also differences in the system of double reading and the method of tie break, in the number of views, in the percentage of clinical investigations and/or the adaptation of the BI-RADs, which greatly hampers comparisons among studies.

## Conclusion

In conclusion, the results obtained in the present study are in line with those of the most recent publications, in which radiologists' experience depends on multiple factors; therefore experience-related variables should not be interpreted in isolation.

There may be an optimal combination of experience and volume that is required to achieve reasonable performance, but greater experience and volume may not contribute to greater improvement. The danger of the volume argument is that even if an association is found between better performance and higher volumes, this association may not be causal. Higher volume radiologists may have better equipment, better feedback loops, etc. that could make them appear to be better readers. What is needed is a demonstration that performance actually improves over time with each mammogram read within the practice of individual radiologists.

## Competing interests

The authors declare that they have no competing interests.

## Authors' contributions

The authors certify that they have made the following contributions to this manuscript: EM: Study concepts, statistical analysis, interpretation, manuscript drafting and literature research. FM: Study concepts, interpretation and manuscript drafting. FF: Study concepts, interpretation and manuscript drafting. MTM: Study concepts, interpretation and manuscript drafting. XC: Study concepts, interpretation and manuscript drafting.

## Pre-publication history

The pre-publication history for this paper can be accessed here:



## References

[B1] Elmore JG, Wells CK, Lee CH, Howard DH, Feinstein AR (1994). Variability in radiologists' interpretations of mammograms. N Engl J Med.

[B2] Beam CA, Layde PM, Sullivan DC (1996). Variability in the interpretation of screening mammograms by US radiologists. Findings from a national sample. Arch Intern Med.

[B3] Carney PA, Elmore JG, Abraham LA, Gerrity MS, Hendrick RE, Taplin SH, Barlow WE, Cutter GR, Poplack SP, D'Orsi CJ (2004). Radiologist uncertainty and the interpretation of screening. Med Decis Making.

[B4] Bull AR, Campbell MJ (1991). Assessment of the psychological impact of a breast screening programme. Br J Radiol.

[B5] Lampic C, Thurfjell E, Bergh J, Sjoden PO (2001). Short- and long-term anxiety and depression in women recalled after breast cancer screening. Eur J Cancer.

[B6] Castells X, Molins E, Macià F (2006). Cumulative false positive recall rate and association with participant related factors in a population based breast cancer screening programme. J Epidemiol Community Health.

[B7] Theberge I, Hebert-Croteau N, Langlois A, Major D, Brisson J (2005). Volume of screening mammography and performance in the Quebec population-based Breast Cancer Screening Program. CMAJ.

[B8] Barlow WE, Chi C, Carney PA, Taplin SH, D'Orsi C, Cutter G, Hendrick RE, Elmore JG (2004). Accuracy of screening mammography interpretation by characteristics of radiologists. J Natl Cancer Inst.

[B9] Elmore JG, Miglioretti DL, Carney PA (2003). Does practice make perfect when interpreting mammography? Part II. J Natl Cancer Inst.

[B10] Kan L, Olivotto IA, Warren Burhenne LJ, Sickles EA, Coldman AJ (2000). Standardized abnormal interpretation and cancer detection ratios to assess reading volume and reader performance in a breast screening program. Radiology.

[B11] Elmore JG, Wells CK, Howard DH (1998). Does diagnostic accuracy in mammography depend on radiologists' experience?. J Womens Health.

[B12] Perry N, Broeders M, de Wolf C, Törnberg S, Holland R, von Karsa L, Puthaar E, eds (2006). European Commission. European Guidelines for Quality Assurance in Breast Cancer Screening and Diagnosis.

[B13] Baker JA, Kornguth PJ, Floyd CE (1996). Breast imaging reporting and data system standardized mammography lexicon: observer variability in lesion description. AJR Am J Roentgenol.

[B14] Lehman C, Holt S, Peacock S, White E, Urban N (2002). Use of the American College of Radiology BI-RADS guidelines by community radiologists: concordance of assessments and recommendations assigned to screening mammograms. AJR Am J Roentgenol.

[B15] Smith-Bindman R, Chu P, Miglioretti DL, Quale C, Rosenberg RD, Cutter G, Geller B, Bacchetti P, Sickles EA, Kerlikowske K (2005). Physician predictors of mammographic accuracy. J Natl Cancer Inst.

[B16] Esserman L, Cowley H, Eberle C, Kirkpatrick A, Chang S, Berbaum K, Gale A (2002). Improving the accuracy of mammography: volume and outcome relationships. J Natl Cancer Inst.

[B17] Beam CA, Conant EF, Sickles EA (2003). Association of volume and volume-independent factors with accuracy in screening mammogram interpretation. J Natl Cancer Inst.

[B18] Sharyl N, Ball J, Institute of Medicine (2005). Improving interpretive performance in mammography. Improving breast imaging quality standards.

[B19] Quality Assurance Guidelines for Radiologists. http://www.cancerscreening.nhs.uk/breastscreen/publications/nhsbsp59.html.

[B20] Clark R (2005). Re: Accuracy of screening mammography interpretation by characteristics of radiologists. J Natl Cancer Inst.

[B21] Smart CR, Byrne C, Smith RA, Garfinkel L, Letton AH, Dodd GD, Beahrs OH (1997). Twenty-year follow-up of the breast cancers diagnosed during the Breast Cancer Detection Demonstration Project. CA Cancer J Clin.

[B22] Kerlikowske K, Grady D, Barclay J, Frankel SD, Ominsky SH, Sickles EA, Ernster V (1998). Variability and accuracy in mammographic interpretation using the American College of Radiology Breast Imaging Reporting and Data System. J Natl Cancer Inst.

[B23] Smith-Bindman R, Ballard-Barbash R, Miglioretti DL, Patnick J, Kerlikowske K (2005). Comparing the performance of mammography screening in the USA and the UK. J Med Screen.

[B24] Yankaskas BC, Klabunde CN, Ancelle-Park R, Renner G, Wang H, Fracheboud J, Pou G, Bulliard JL (2004). International comparison of performance measures for screening mammography: can it be done?. J Med Screen.

[B25] Smith RA, Vainio H, Bianchini F (2002). Breast cancer screening. IARC handbooks of cancer prevention.

